# The cell-to-cell coordination between activated T cells and CpG-stimulated macrophages synergistically induce elevated levels of IL-10 via NF-κB1, STAT3, and CD40/CD154

**DOI:** 10.1186/1478-811X-11-95

**Published:** 2013-12-13

**Authors:** Denada Dibra, Shulin Li

**Affiliations:** 1 Department of Pediatrics, The University of Texas MD Anderson Cancer Center, 1515 Holcombe Blvd., Houston, TX 77030, USA; 2 The University of Texas Graduate School of Biomedical Sciences at Houston, The University of Texas MD Anderson Cancer Center, 1515 Holcombe Blvd., Houston, TX 77030, USA

**Keywords:** IL-10, IL27p28 (IL-30), NF-κB1, STAT3, CD40/CD154, CD4^+^ T cells, pERK, F4/80 cells

## Abstract

**Background:**

Studies into the regulation of interleukin-10 (IL-10), have focused only on the molecular or single-cell level. The cues that induce IL-10 in the context of cell-to-cell communication are scarce. To fill this gap, this study elucidates the cell-to-cell interaction dependent regulation of IL-10.

**Results:**

The simultaneous activation of CD4^+^ T cells via CD3/CD28 and stimulation of macrophages via CpG and their intercellular communication with each other in the same microenvironment is necessary to induce a synergistic expression of IL-10. NF-κB1, ERK, and STAT3 are positive regulators of this cell-to-cell communication mediated molecular change of IL-10 induction. Strikingly, the activation of CD40/CD154 signaling is a negative regulator of IL-10 levels by CD3/CD28/CpG.

**Conclusions:**

These findings are of prominence as CD3/CD28/CpG treatment can induce the anti-inflammatory cytokines IL-10 and IL-30, and the activation or inhibition of the CD40/CD154 acts as molecular rheostat of the expression of IL-10 or IL-30. More importantly, this not only serves as an example of IL-10 regulation at the cellular via coordination of two signals from two cell types, but these findings also lay the molecular and cellular groundwork for future studies to investigate how to manipulate IL-10 or IL-30 production during inflammation, cancer, or autoimmune diseases.

## Background

The immune response has evolved to clear pathogens and, subsequently, to limit this immune response to protect the host from damage, therefore, a successful response must balance these pro- and anti-inflammatory signals. Particularly, IL-10 is a major anti-inflammatory cytokine that prevents autoimmune and inflammatory diseases [1,2]. Additionally, IL-10 has been shown to be a feedback regulator of Th1, Th2, and allergenic immune responses [3,4]. This cytokine is produced by different immune cell types, including B cells, macrophages, mast cells, neutrophils, dendritic cells (DC), and several T cell subsets (including Th1, Th17, Foxp3^+^ Tregs, and regulatory type 1 cells Tr1) [5-8]. The production of IL-10 from CD4^+^ T cells, CD8^+^ T cells, and myeloid cells (e.g., DC) have all been shown to play important roles in regulating immunopathology in different infectious disease.

Different cues within the microenvironment regulate the IL-10 expression in a cell-specific manner. This observation is of importance as IL-10 expression by different cells, namely effector CD4^+^ or regulatory T (T_reg_) cells, can have different roles in the same infection. For example, in *L. major* infections IL-10 from effector Th1 cells is necessary for suppression of inflammatory responses during acute infection, whereas IL-10- producing antigen-specific Foxp3^+^ CD4^+^ T cells (T regs) suppress the clearance of the parasite by the effector CD4^+^ T cells [9,10]. Additionally, the molecular mechanism for the upregulation of IL-10 differs among cell types. For example, in Th1 cells, MAF and SMAD4 are key IL-10 transcription factors; however, in Th2 cells, GATA3, Jun, and MAF are specific transcription factors for IL-10 expression, while STAT3 or STAT1 are the important factors for IL10 in Th17 expression [11-14].

While most of the regulation of IL-10 expression described in the literature is limited to the molecular and single cell level within a specific type of immune cell, the cell-to-cell interaction-dependent regulation has not been examined. Understanding the cell-to-cell communication in regulating IL-10 levels is important, as it not only replicates the inter-cellular communication that occurs in vivo, but also integrates different cues within the microenvironment to account for IL-10 levels globally. Obtaining this information can lead to more effective interventions during inflammatory and pathogenic immunopathologies. Here we demonstrate that simultaneous activation of two types of cells, CD4^+^ T cells via CD3/CD28 and CpG-stimulated macrophages and their interaction in the same microenvironment is crucial to induce robust expression of IL-10. Furthermore, this upregulation of IL-10 occurs via NF-κB1 and STAT3 activation. This work is of importance as it provides an example of IL-10 regulation at the cell-to-cell and molecular level via coordination of two signals from two cell types.

## Results

**Figure 1:**
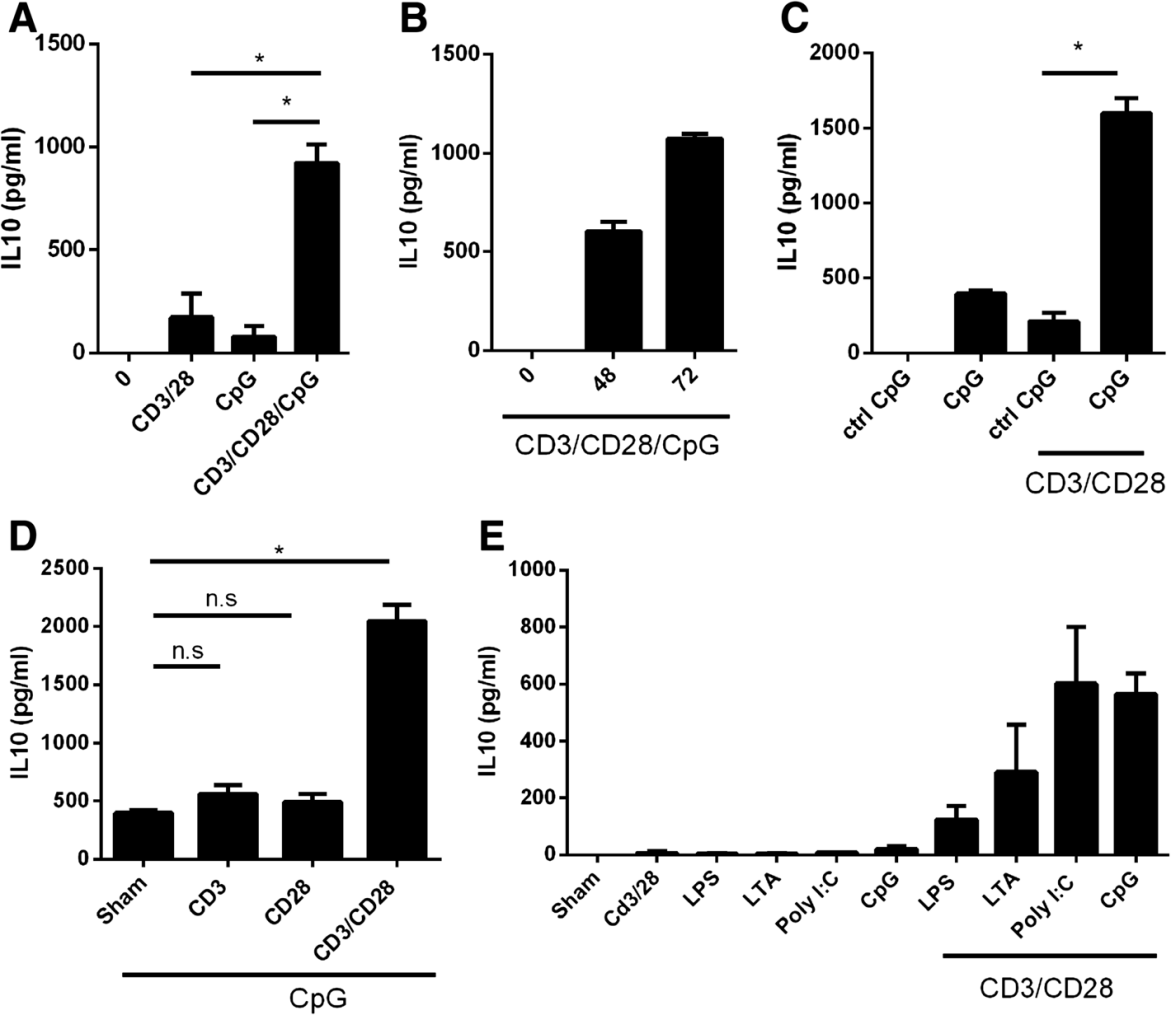
**Coadministration of CD3/CD28 and CpG induce robust amounts of IL-10 expression in splenocytes when compared to either signal alone. (A)** Splenocytes were treated with CpG, CD3/CD28, CD3/CD28/CpG, or left untreated for 72 hours and analyzed for IL0 production by ELISA. **(B)** Splenocytes were treated with CD3/CD28/CpG for 0, 48, or 72 hours, and IL-10 levels were measured by ELISA. **(C)** Supernatants of splenocytes treated with either CpG or control ODN (ctrl CpG) in the absence or presence of CD3/CD28 for 72 hours were analyzed for IL-10 production by ELISA. **(D)** Splenocytes were treated with CpG in the presence or absence of various T cell-activating antibodies (CD28, CD3, or CD3/CD28) for 72 hours, and IL-10 expression in the supernatants was measured via ELISA. **(E)** Supernatants from splenocytes treated as indicated for 72 hours were analyzed for IL-10 production by ELISA. *, P <0.05. N = 3.

The essential transcription factor(s) or pathway(s) that activates IL-10 expression is dependent on the type of both the cell and stimuli [11,15]. While many studies have attempted to understand the regulation of IL-10 in a specific cell type through a specific stimulus, the role of cell-to-cell communication or interaction in the induction of IL-10 is largely unknown. To test the coordination of different types of immune cells in inducing IL-10, two signals that stimulate different cell types were independently or simultaneously applied to the cell mixtures (splenocytes). The first signal, comprised of CD3 and CD28 (CD3/CD28), mimics the first and second signals that activate T cells and can induce moderate amounts of IL-10. The other stimulation signal was CpG which activates cells via the TLR9 receptor present on many types of cells but primarily on antigen presenting cells (APCs) such as macrophages, DC, and B cells, and can induce IL-10 expression as well. The rationale for using splenocytes was that this cell mixture represents a variety of immune cells, and additive or synergistic effects of different stimuli can be observed. Additionally, these two sets of signals, CD3/CD28 plus CpG (CD3/CD28/CpG), synergistically induces high levels of the anti-inflammatory cytokine IL-30 [16], therefore suggesting that the same phenomenon may occur in IL-10 regulation. Indeed, the treatment of cells with the CD3/CD28/CpG combination induced synergistic expression of IL-10 in splenocytes; however, treatment of cells with either CD3/CD28 or CpG alone induced a relatively small increase in IL-10 production (Figure 1A). Kinetic studies showed that the highest induction of IL-10 via CD3/CD28/CpG occurs 72 hours after treatment (Figure 1B). CD3/CD28/CpG induced significantly higher IL-10 levels compared to either signal alone, whereas treatment with CD3/CD28 and control isogenic CpG (in which CpG was switched to GpC) did not effectively raise IL-10 levels (Figure 1C). High levels of IL10 levels were not due increase in cell proliferation by the combination treatment (Additional file 1: Figure S1). These data revealed that either CD3/CD28 or CpG alone induces a moderate amount of IL-10 expression, and the combination of these two treatments synergistically raises IL-10 levels. This result suggests that simultaneous activation of both T cells and TLR9^+^ cell signaling is crucial for inducing robust IL-10 expression.

To determine whether both primary and secondary signals are required to coordinate with CpG to boost IL-10 expression, splenocytes were activated in the presence of CpG with agonist antibodies for the T cell activators CD28, CD3, or both CD3 plus CD28. Interestingly, efficient activation of T cells with both the primary (CD3) and secondary signals (CD28) and not with either signal alone is able to induce high levels of IL-10 production (Figure 1D). To determine whether this effect is specific for TLR9, various TLR ligands were used in the presence of CD3/CD28 antibodies. The enhanced expression of IL-10 in coordination with T cell activation is not specific only to TLR9 since stimulation with other TLR ligands induced expression of IL-10 (Figure 1E). This observation suggests that many different stimuli from pathogens to APCs in the presence of activated T cells induce high levels of IL-10, confirming that many activation pathways converge at IL-10 to protect the host against inflammation.

**Figure 2:**
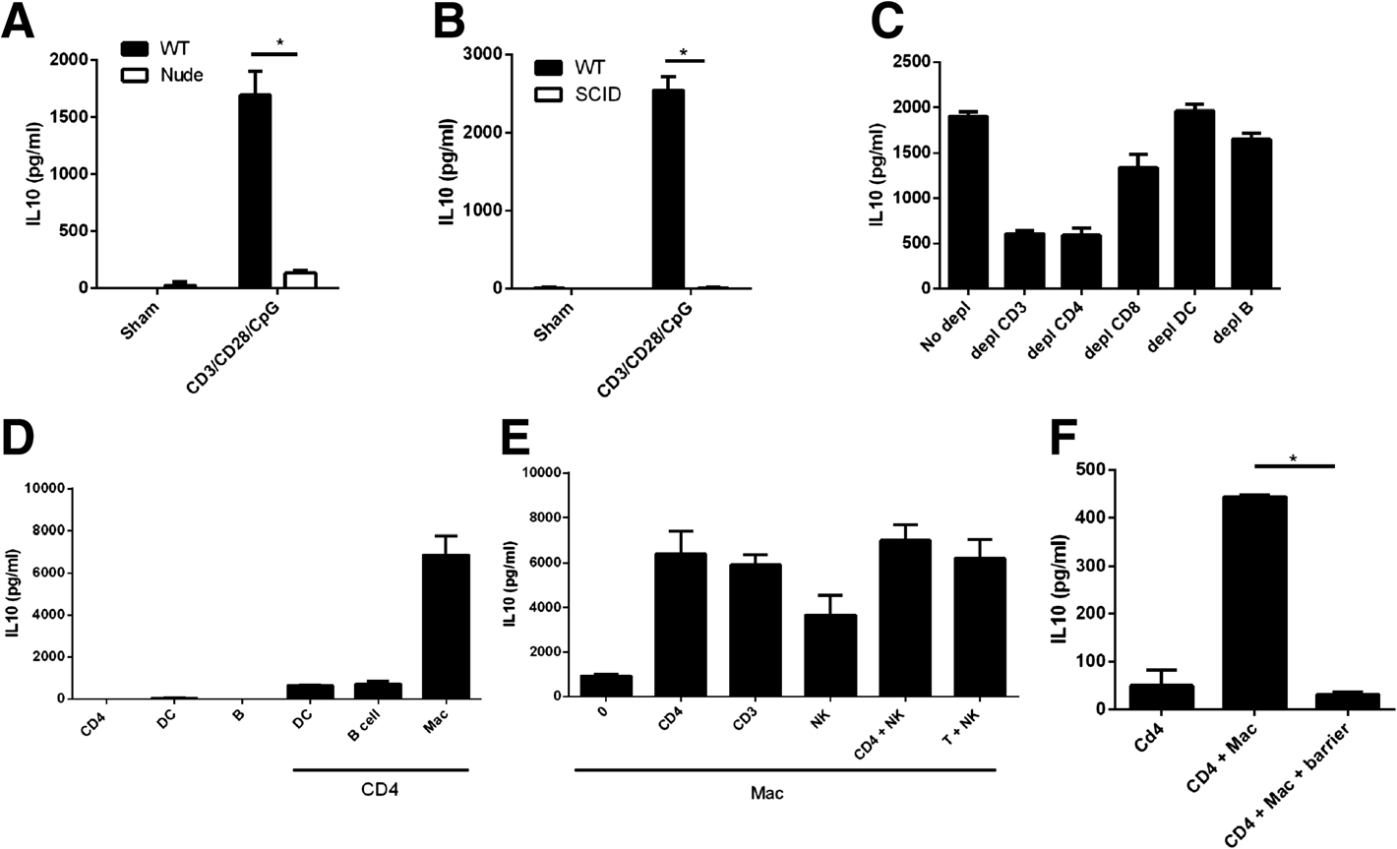
**Coordination between T cells and macrophages induces the highest expression of IL-10 in a cell-contact dependent manner. (A, B)** Supernatants from wild type and nude (**A**, Balb/c background) or wild type and SCID (**B**, C3H background) splenocytes were treated with CD3/CD28/CpG for 72 hours and then analyzed for IL-10 production by ELISA. **(C)** Non-depleted splenocytes or the ones depleted for CD3, CD4, CD8, DC, and NK cells were treated with CD3/CD28/CpG for 72 hours, and the supernatants were analyzed for IL-10 expression via ELISA. **(D)** Purified splenic B cells, DC, CD4^+^ T cells, or peritoneal macrophages were coincubated in the presence or absence of purified CD4^+^ T cells, treated with CD3/CD28/CpG for 72 hours and analyzed for IL-10 expression in the supernatants by ELISA. **(E)** Peritoneal macrophages were coincubated in the presence or absence of purified NK, whole T (CD3^+^ T), CD4^+^ T, or a combination of CD4^+^ T and NK cells; treated with CD3/CD28/CpG for 72 hours; and analyzed for IL-10 expression in the supernatants via ELISA. **(F)** Peritoneal macrophages were coincubated with purified CD4^+^ cells in the presence or absence of a transwell barrier, treated with CD3/CD28/CpG for 72 hours, and analyzed for IL-10 expression in the supernatant via ELISA. *, P <0.05. N = 3.

Immunocompromised nude mice were used to further confirm the role of T cells, and the requirement of T and other immune cell interaction in inducing high levels of IL-10. As predicted, the absence of functional T cells eliminated the CD3/CD28/CpG-mediated synergistic induction of IL-10 (Figure 2A). Similar results were also seen in immunodeficient SCID mice (Figure 2B). Furthermore, depletion of CD4 cells, but not CD8, DC, or B cells, inhibited the most IL-10 expression (Figure 2C), suggesting that CD4^+^ T cell activation via CD3/CD28 is essential for maximal induction of IL-10.

The initial hypothesis was that interaction of two different types of immune cells via the CD3/CD28/CpG signal is needed to maximally upregulate IL-10. From the depletion studies, we saw that CD4^+^ T cells play a crucial role. Amongst the antigen presenting cells tested, depletion of DC and B cells did not significantly lower IL-10, suggesting that macrophages might be the key interacting cells. To confirm this hypothesis, we performed a series of cell mixture studies using different antigen presenting cells. The purified CD4^+^ T cells were co-incubated with various types of APC, such as B cells, macrophages, and DC and the resulting supernatants were tested for IL-10 expression. We found that a mixture of macrophages and CD4^+^ T cells yields a high level of IL-10 expression upon exposure to CD3/CD28/CpG (Figure 2D), whilst B cells and DC only play a minor role in the induction of IL-10. These results clearly indicate that the interaction between CD4^+^ T cells and macrophages are required to induce a high level of IL-10 expression in the presence of this combination of signals.

To confirm that CD4^+^ T cells and macrophages interact to induce high levels of IL-10 expression, macrophages were reconstituted with CD4^+^ T cells, CD4^+^ T and NK cells, or whole T cells (CD3^+^ cells). As expected, reconstitution of macrophages with CD4^+^ T cells raised the IL-10 levels following CD3/CD28/CpG treatment (Figure 2E). Additionally, NK cells boosted the level of CD3/CD28/CpG-induced IL-10 expression, yet the overall expression levels were much lower when compared to those of CD4^+^ T cells. These differences suggest that CD4^+^ T cells play the major role in IL-10 biology.

T cells can activate macrophages via cell contact. To determine whether macrophages and T cells need physical contact to induce IL10 upregulation, macrophages and CD4^+^ T cells were coincubated in the presence or absence of a transwell barrier, treated with CD3/CD28/CpG, and analyzed for IL10 protein expression. In the presence of a barrier, CD3/CD28/CpG failed to induce any IL10 expression, suggesting that the expression of IL10 requires direct cell-to-cell contact between macrophages and CD4^+^ T cells (Figure 2F) suggesting that cell-cell communication is needed to upregulate IL10.

**Figure 3:**
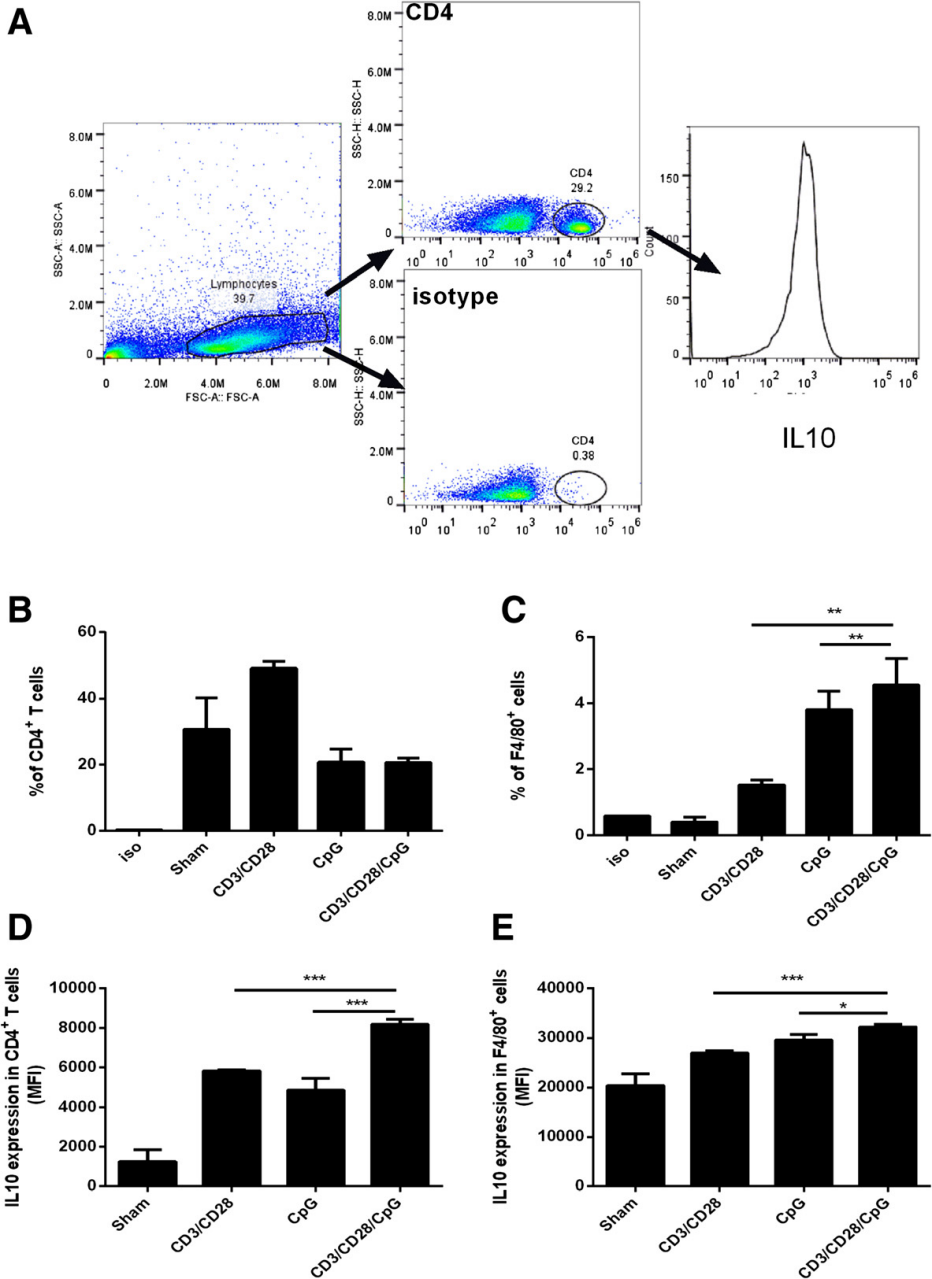
**CD3/28/CpG induces IL-10 production in both macrophages and CD4**^**+ **^**T cells. (A)** Diagram of FACS analysis. **(B, C)** Splenocytes left untreated or treated with CpG, CD3/CD28, CD3/CD28/CpG for 72 hours and number of CD4 **(B)** or F4/80 **(C)** were measured via FACS. **(C, D)** Median fluorescence intensity of IL10 in the CD4^+^**(D)** or F4/80^+^**(E)** gated cells. *, P <0.05. N = 3.

While the data above suggests that interaction between both macrophages and T cells is needed to effectively produce IL-10, the cell source that produces IL-10 via the combination treatment is not known. To understand this origin of IL-10, splenocytes were treated with CD3/CD28, CpG, or the combination treatment, and these cells were stained via flow cytometry for IL-10 expression either in CD4^+^ cells or macrophages (via the macrophage specific antibody F4/80) (Figure 3A). Interestingly, the combination treatment induces a higher number of macrophages (Figure 3C), while it has no effect on the percentage of CD4^+^ T cells (Figure 3B). Meanwhile, the CD3/CD28/CpG combination treatment significantly increases IL10 expression in CD4 cells (Figure 3D), while it induces only a modest increase of IL10 in macrophages (Figure 3E). Aside from inducing higher levels of IL10 per cell in the CD4 cell population, the combination treatment CD3/CD28/CpG additionally results in a reduction of Treg cells *in vitro* (Additional file 2: Figure S2).

Therefore, this data suggests that both CD4^+^ T cells and macrophages contribute to increased IL-10 expression.

**Figure 4:**
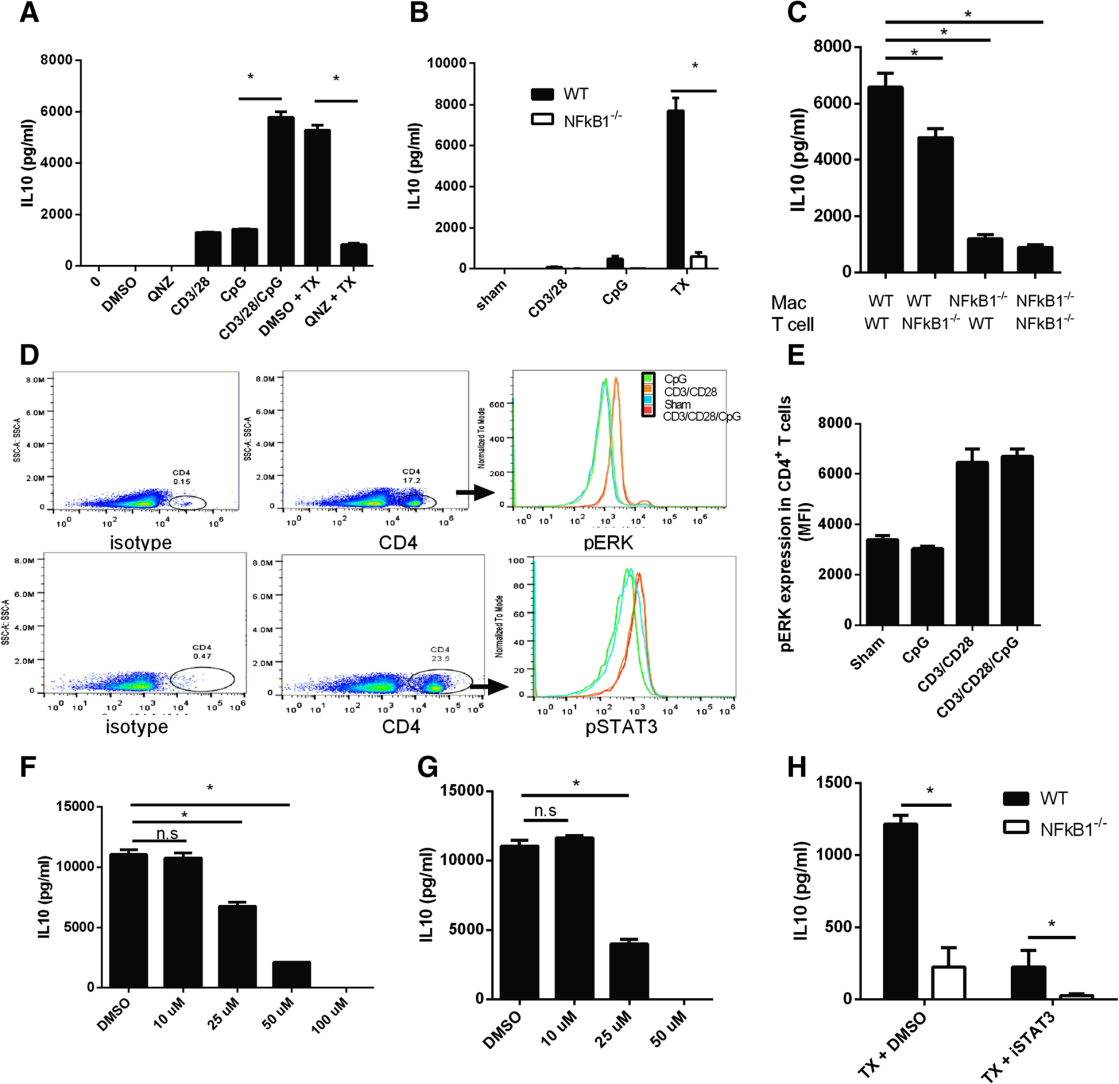
**CD3/CD28/CpG regulates IL-10 via activation of NF-κB1,pERK, and STAT3. (A)** Splenocytes were treated as indicated for 72 hours and IL-10 levels were measured by ELISA. **(B)** Supernatants from wild type or NF-κB1^-/-^ splenocytes treated with CD3/CD28, CpG, or TX (CD3/CD28/CpG) for 72 hours were analyzed for IL-10 production by ELISA. **(C)** Peritoneal macrophages from wild type or NF-κB1^-/-^ mice were coincubated with purified CD4^+^ T cells from either wild type or NF-κB1^-/-^ mice, treated with CD3/CD28/CpG for 72 hours and the supernatants were measured for IL-10 expression by ELISA. **(D)** Splenocytes treated with CD3/CD28, CpG, or TX (CD3/CD28/CpG) for 72 hours were stained for CD4 and pERK or CD4 and pSTAT3. Histograms show pERK or pSTAT3 levels from gated T cells. **(E)** Quantification of median fluorescence intensity of pERK levels in CD4 cells (N = 3). **(F)** Splenocytes were treated with ERK inhibitor U0126 at various doses for 72 hours in presence of CD3/CD28/CpG, and IL-10 levels were measure by ELISA. **(G)** Splenocytes were treated with STAT3 inhibitor NSC 74859 at various doses for 72 hours in presence of CD3/CD28/CpG, and IL-10 levels were measure by ELISA. **(H)** Wild type or NF-κB1^-/-^ splenocytes in presence or absence of STAT3 inhibitor (STAT3i, 50 μM) were treated with CD3/CD28/CpG for 72 hours and analyzed for IL-10 production by ELISA. *, P <0.05. N = 3.

While it is understood the T and macrophage coordination needed to induce IL-10, the transcription factors involved in driving IL-10 expression by the cell-to-cell communication has not been investigated. Activation of transcription factors NF-κB1 and STAT3 play a crucial role in regulation of IL-10 in a single cell system [17,18]. Importantly, NF-κB1 has been shown to transactivate the promoter of IL-10. To determine whether CD3/CD28/CpG induces IL-10 through modulation of NF-κB1, splenocytes were treated with CD3/CD28/CpG in the presence or absence of NF-κB inhibitor (Figure 4A). The presence of the NF-κB inhibitor significantly inhibited IL-10 expression. To further prove that the absence of NF-κB indeed affects IL-10 expression, splenocytes defective in the NF-κB pathway (NF-κB1^-/-^ splenocytes) were treated with the combination treatment. Indeed, the absence of NF-κB1 hinders IL-10 expression by CD3/CD28/CpG by 8 fold (Figure 4B). Indeed, since both NF-κB1 activation and the cell communication plays a major role in regulating IL-10 expression, it is important to understand whether NF-κB1 signaling primarily in macrophages or T cells is needed. Interesting, the absence of NF-κB1 in macrophages plays a major role in IL-10 regulation, but the absence of NF-κB1 signaling in T cells only plays a minor role (Figure 4C).

Since it is known that NF-κB1 is the crucial transcription factor to induce IL10 in macrophages, we sought to determine the molecular mechanism that was activated in CD4^+^ T cells. Both pERK and pSTAT3 were upregulated in CD4^+^ T cells by the combination treatment CD3/CD28/CpG when compared to untreated samples (Figure 4D, E) at 72 hours. Meanwhile, CD3/CD28/CpG and CD3/CD28 exert the same effect on T cells, and induce similar levels of either pSTAT3 or pERK (Figure 4D). Time course study showed that pERK was induced at a similar trend at 10, 30 min, and 1 hour as it was at 72 hours (Additional file 3: Figure S3). To verify that indeed these two transcription factors are involved IL10 induction by CD3/CD28/CpG, we used chemical inhibitors to reverse the phenotype. Indeed, ERK or STAT3 inhibition significantly affects IL-10 levels in a dose-dependent manner respectively (Figure 4F, G). Furthermore, inhibition of both NF-κB1 and STAT3 (50 μM) pathways obstructs IL-10 expression to almost undetectable levels (Figure 4H).

**Figure 5:**
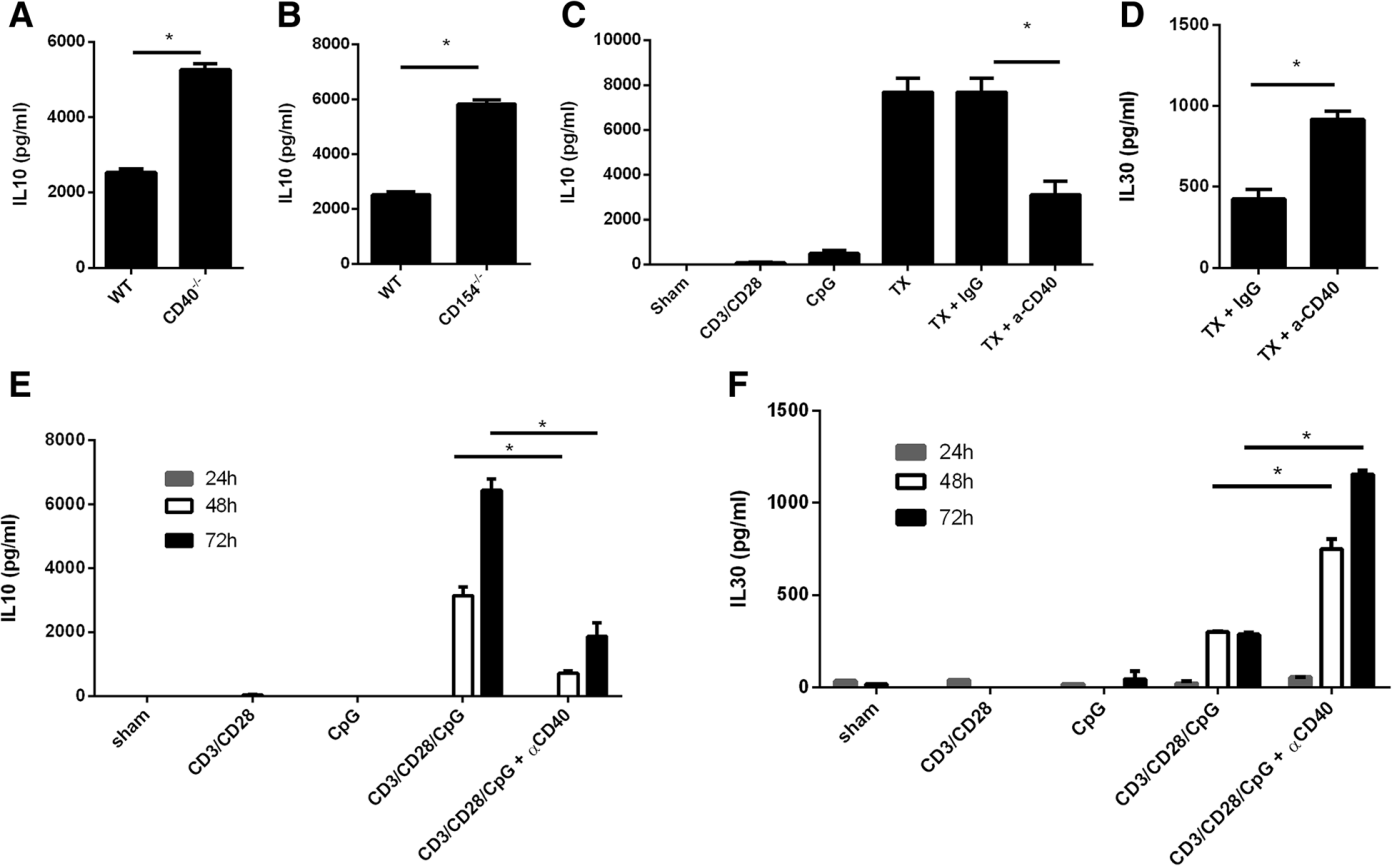
**Activation or inhibition of CD40/CD154 pathway in the presence of CD3/CD28/CpG acts as a switch in the expression of IL-10 or IL-30. (A)** Supernatants from wildtype or CD40^-/-^splenocytes were treated with CD3/CD28/CpG for 72 hours were analyzed for IL-10 production by ELISA. **(B)** Supernatants from wild type or CD154^-/-^ splenocytes treated with CD3/CD28/CpG for 72 hours were analyzed for IL-10 production by ELISA. **(C, D)** Splenocytes from wild type mice were treated with the indicated treatments in the presence of control or anti-CD40 antibodies for 72 hours, and IL-10 **(C)** or IL-30 **(D)** expression was measured in the supernatants using ELISA. **(E, F)** Splenocytes from wild type mice were treated with the indicated treatments in the presence of control or anti-CD40 antibodies for 24, 48, or 72 hours, and IL-10 **(E)** or IL-30 **(F)** expression was measured in the supernatants using ELISA. *, P <0.05. N = 3.

The fact that both IL-10 and IL-30 are induced via the CD3/CD28/CpG provokes the question of whether the combination treatment can preferentially induce one cytokine over the other under different contextual clues. If so, which pathway is involved in this process? CD154 is one of the ligands on the T cell membrane that can bind to and activate the CD40 receptor on macrophage. Additionally, this pathway is critical to raise IL-30 expression when combined with CD3/CD28/CpG [16]. To evaluate the involvement of CD40/CD154 pathway during CD3/CD28/CpG-mediated regulation of IL-10, we compared IL-10 levels induced by CD3/CD28/CpG in wild type, CD40^-/-^, and CD154^-/-^ splenocytes (Figure 5A and Figure 5B). Opposite to IL30 induction by CD3/CD28/CpG, the absence of CD40 or CD154 raised IL-10 expression by more than 2 fold compared to wild type, indicating that the CD40/CD154 pathway is a negative rather than positive regulator. Furthermore, wild type splenocytes treated with agonist CD40 antibody (anti-CD40) lowered the IL-10 production by one half (Figure 5C). These results confirm the data from the CD40^-/-^ and CD154^-/-^ knockout splenocytes. Meanwhile, agonist anti-CD40 antibody in the presence of the CD3/CD28/CpG treatment significantly raises IL-30 levels by at least two fold (Figure 5D). To exclude the possibility that the observed effects are specific to the time point chosen, we tested different time points. Indeed, this data showed that activation of CD40 in presence of CD3/CD28/CpG reduced IL10 and increased IL30 in all the tested time points (Figure 5E, F), therefore suggesting that activation of CD40 during the combination treatment acts as a molecular rheostat in the regulation of IL10 and IL30 [16].

**Figure 6:**
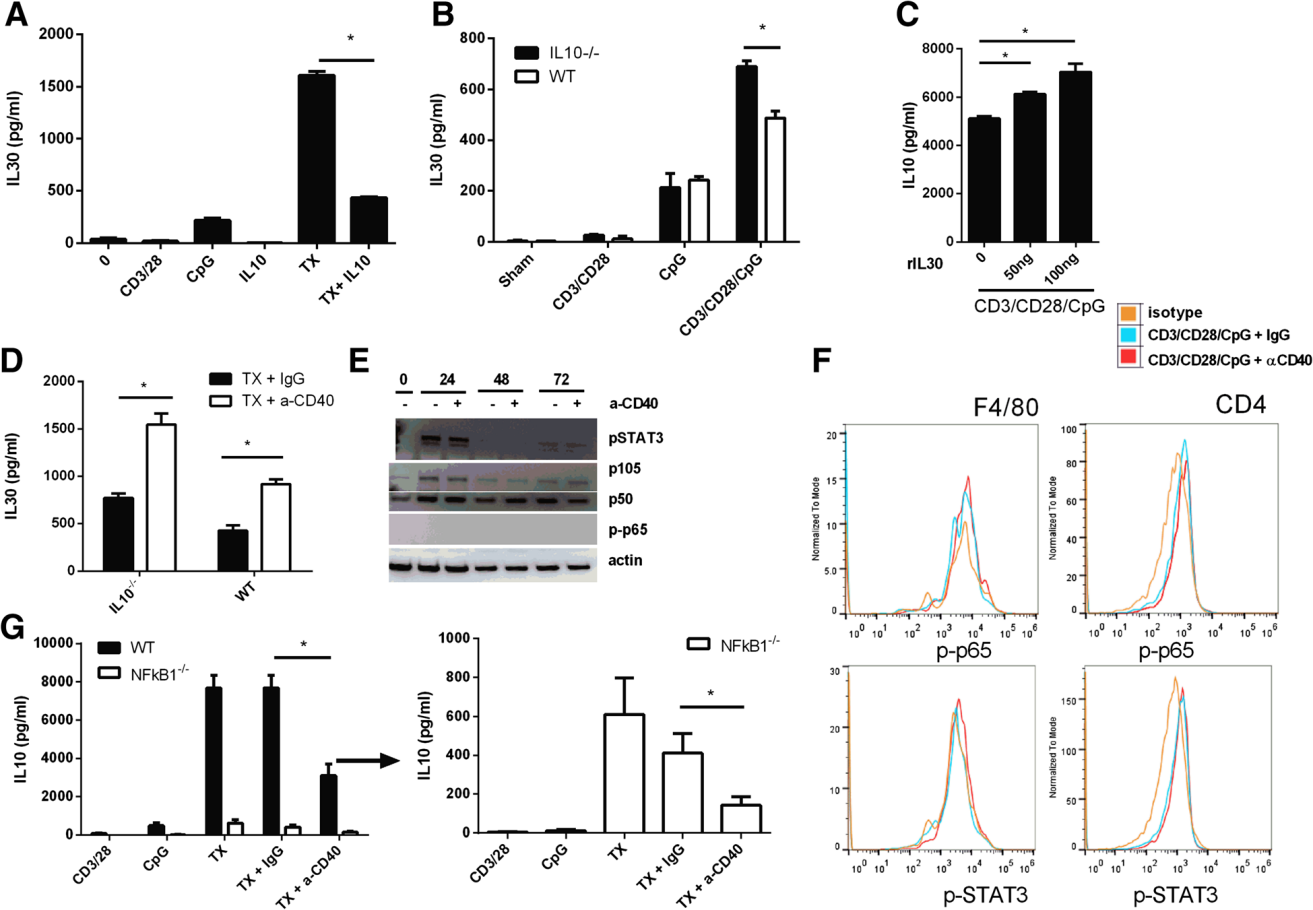
**Regulation of IL-10 and IL-30 via activation of CD40 signaling. (A)** Supernatants from splenocytes treated with CpG, CD3/CD28, TX (CD3/CD28/CpG), in the presence or absence of rIL-10 for 72 hours were analyzed for IL-30 production by ELISA. **(B)** Wild type or IL-10^-/-^ splenocytes were treated as indicated for 72 hours, and IL-30 levels were measured by ELISA. **(C)** Splenocytes were treated with CD3/CD28/CpG in the presence of recombinant IL30 at 50 and 100 ng/ml for 72 hours, and IL-10 levels were measured by ELISA. **(D)** Wild type or IL-10^-/-^ splenocytes were treated with CD3/CD28/CpG for 72 hours in the presence of control or anti-CD40 antibodies, and IL-30 levels were measured by ELISA. **(E)** Harvested pellets of splenocytes treated with anti-CD40 or control antibody for the indicated time points were probed for pSTAT3, p-p65, p50/105, and actin. **(F)** Splenocytes were treated with CD3/CD28/CpG in the presence or absence of anti-CD40 activating antibody and the levels of p-p65 and p-STAT3 in the CD4^+^ T cells or macrophages were measured via flow cytometry. **(G)** Supernatants from wild type or NF-κB1^-/-^ splenocytes treated with CpG, CD3/CD28, TX (CD3/CD28/CpG), or CD3/CD28/CpG in the presence of activating anti-CD40 for 72 hours were analyzed for IL-30 production by ELISA. *, P <0.05. N = 3.

Our previous study shows that CD3/CD28/CpG induces IL-30, and this study shows that the same stimuli induce IL10 expression [16]. An intriguing question is whether IL-10 regulates IL30 via the CD3/CD28/CpG combination. To answer this question, splenocytes were treated with the combination signal in the presence or absence of IL-10. In the presence of recombinant IL-10, IL-30 expression levels induced by CD3/CD28/CpG were significantly inhibited (Figure 6A), suggesting that IL-10 negatively regulates IL-30 expression. Additionally, in IL-10^-/-^ splenocytes, CD3/CD28/CpG induces higher levels of IL-30 when compared to wildtype counterparts (Figure 6B). These data suggest that the combination treatment induces high levels of both IL-10 and IL-30, but IL-10 also serves as a negative inhibitor of IL-30. However, recombinant IL30 boosts IL10 levels in a dose-dependent manner (Figure 6C).

Ligation of CD40 (via the agonist antibody) inhibits IL-10 upregulation by CD3/CD28/CpG (Figure 4), while it promotes IL-30 expression [16]. This observation provokes the question whether IL-10 or activation of CD40 signaling depend on each other to alter IL-30 expression. Indeed, even in the presence or absence of IL-10, anti-CD40 stimulation upregulates IL-30 expression (Figure 6D). These results suggest that both IL-10 and CD40 can regulate IL-30 expression via two separate mechanisms.

The activation of the CD40 pathway via the agonist antibody lowers IL-10 induction while the activation of NF-κB1 and STAT3 raises IL-10 expression. One possibility is that the activation of CD40 lowers NF-κB1 or STAT3 activation over time, therefore affecting IL-10 expression. To test this hypothesis, splenocytes were treated in the absence or presence of agonist anti-CD40 antibody over time and probed for the phosphor-STAT3 or NF-κB1 p65, or NF-κB p50/105. There was no difference in the levels of STAT3 activation or the total levels of NF-κB p50/105 by the engagement of CD40 in splenocytes (Figure 6E). We also tested whether these specific transcription factors were upregulated in specific cell populations, such as CD4^+^ T cell or macrophages. Indeed, no differences in the levels of p-p65 or pSTAT3 were observed between samples treated with CD3/CD28/CpG in either the presence or absence of CD40 (Figure 6F). Additionally, even in absence of NF-κB1 signaling, anti-CD40 can further inhibit IL-10 expression levels (Figure 6G). Because IL10 and IL30 also regulate each other and CD40/CD154 acts a rheostat in the regulation of IL10 or IL30, one possibility is that these cytokines affect the CD40/CD154 pathway. To test this hypothesis, splenocytes were treated with rIL10 or rIL30 and the levels of CD154 in CD4+ T cells and CD40 levels on macrophages were tested. Neither rIL0 nor rIL30 altered CD40 or CD154 in macrophages or T cells, respectively (Additional file 4: Figure S4). Overall, these data demonstrate that CD40, NF-κB1, and STAT3 pathway can regulate IL-10 independently of each other.

## Discussion

Owing to the vital role of IL-10 in the immune response and the link between defective IL-10 production and certain autoimmune and inflammatory diseases as well as cancer, an understanding of the molecular and cellular mechanisms that regulate this cytokine is critical [1,2]. Extensive studies have investigated the regulation of IL-10 within single immune cell subsets and shown that IL-10 regulates its own expression through positive and negative feedback loops; however, the cell-to-cell interaction dependent regulation of IL-10 within diverse immune cell populations is largely unknown. In this study we discovered that cell-to-cell communication via the two signaling pathways CD3/28 and CpG in CD4^+^ T cells and macrophages, respectively, is the key to effectively induce IL-10 expression. We further delved into the mechanism, and we discovered that NF-κB1 and STAT3 are positive regulators, while CD40/CD154 signaling is a negative regulator of IL-10 expression via the combination treatment CD3/CD28/CpG (Figures 4 and 5).

The activation or inhibition of the CD40/CD154 signaling pathway acts as a switch to determines whether the CD3/CD28/CpG treatments upregulate IL-10 or IL-30. The activation of this pathway is a negative regulator of IL-10 in the presence of the combination treatment CD3/CD28/CpG. Three lines of evidence confirm that CD40/CD154 plays a negative role in the regulation of IL-10 in this study (Figure 5). First, the absence of CD40 increases IL-10 CD3/CD28/CpG-induced expression levels. Second, the effect on the expression of IL-10 by the absence of CD154 mirrors the data seen in CD40^-/-^ splenocytes and shows that absence of this pathway promotes IL-10 production. Third, the ligation of CD40 via the agonist CD40 antibody also inhibits IL-10 expression levels. Interestingly, the activation of CD40 can inhibit IL-10 even in the absence of NF-κB1 which highlights the complexity of IL-10 regulation in these models. The role of CD40 in modulating IL-10 expression in the presence of the CD3/CD28/CpG (lowers IL-10 levels) is different from simply activating CD40 directly on macrophages without any other signals (raises IL-10 levels) [19]. These findings are of interest as they display that the same pathway under different circumstances can have a different outcome in the regulation of IL-10. Additionally, the activation of CD40/C154 in the presence of CD3/CD28/CpG differently regulates IL-30, in which case CD40 ligation actually raises the levels of IL-30 [16]. The implications of these findings are of importance because the cell-to-cell communication via the CD3/CD28/CpG treatment can induce both anti-inflammatory cytokines IL-10 and IL-30 and the activation or inhibition of the CD40/CD154 pathway with CD3/CD28/CpG acts as a switch to determine whether IL-10 or IL-30 is induced.

Here we discovered that NF-κB1 is a crucial factor that integrates the CD3/CD28/CpG stimuli to induce IL-10 by the combination treatment. Likewise, it is the NF-κB1 activation within the macrophages that is primarily needed to effectively induce IL-10 as evidenced by the fact that a lack of NF-κB1 in macrophages but not in T cells significantly hampers IL-10 induction. The involvement of NF-κB1 in the model employed in these studies is in concert with another study showing that NF-κB1 binds to the proximal IL-10 promoter in mouse macrophages upon LPS stimulation [17]. In addition to NF-κB1, the activation of STAT3 also upregulates the IL-10 expression via CD3/CD28/CpG. Furthermore, the inhibition of both STAT3 activation and NF-κB1can further inhibit IL-10 expression to almost undetectable levels, which suggests that STAT3 and NF-κB1 can act independently in regulating IL-10 (Figure 5). Additionally, this data also suggest that these two transcription factors are the crucial ones to regulate IL-10 via the cell-to-cell mediated communication. In summary, NF-κB1, STAT3 and CD40 are important regulators of IL-10 and can raise/lower IL-10 even in the absence of each other, which suggests that the expression of IL-10 is complex and tightly regulated.

Many differences are seen in the activated pathways that lead to expression of IL-10 or IL-30 through CD3/CD28/CpG combination treatment. For instance, the activation of T cells in the presence of CpG is the only signal necessary to induce IL-30, but IL-10 can be induced by bacterial or protozoan remnants in coordination with activated T cells. This difference suggests that IL-10 has wider applications to attenuate an immune response whilst IL-30 is only specific to bacterial wall remnants. Interestingly, IL-10 might regulate IL-30 expression since both these anti-inflammatory cytokines are induced by the combination treatment. Indeed, IL-10 lowers IL-30 expression during the combination treatment CD3/CD28/CpG, as the recombinant IL-10 and the knockout data reveal the same conclusion (Figure 6A, B). These data is of interest as it suggests that this treatment modality induces two different anti-inflammatory cells, IL-10 and IL-30, and at the same time, IL-10 acts as a negative regulator of IL-30, thereby having a “build-in” negative signal. Another explanation could be IL-10 does not regulate IL-30 expression directly but probably indirectly by inhibiting T cell proliferation: IL-10^-/-^ splenocytes proliferate more than the wildtype counterpart, and, hence, produce more positive signal to induce IL-30 expression. So, more experiments are needed to determine whether IL-10 affects IL-30 directly or indirectly.

## Conclusion

In conclusion, we have described a novel cellular mechanism that controls IL-10 production. Data presented here reveal that coordination between an innate immune cell-derived signal and a helper T cell-derived signal is necessary to induce a high level of IL-10 expression via the activation of NF-κB1 and STAT3. Additionally, the activation or inhibition of the CD40/CD154 signaling pathway acts as a molecular rheostat to determines whether the CD3/CD28/CpG treatments upregulate IL-10 or IL-30 These findings lay the groundwork for future studies to investigate how to differentially manipulate IL-10 or IL-30 production during inflammation, cancer, or autoimmune diseases.

## Materials and methods

### Reagents

Vendors for all reagents were as follows: thiol-modified CpG oligodeoxynucleotide (ODN) 1668 or control ODN (Sigma), anti-mouse CD3 (eBioscience), anti-mouse CD28 (Biolegend), activating anti-CD40 (Novus, NBP1-06657), recombinant mouse IL12, IFNγ, and IL-10 (eBioscience), LPS (Sigma), lipoteichoic acid (Invivogen), poly I:C (Invivogen), concanamycin A (Sigma), and rat IgG (eBioscience), QNZ (Cayman Chemicals), U0126 (Sigma Aldrich), and NSC 74859 (SelleckBio), pSTAT3, p-p65, STAT3 (Cell signaling), pERK (Santa Cruz), CD154, FOXP3 (eBioscience), recombinant IL27p28 (IL30, Genscript).

### Cell separation and coincubations

Splenocytes were prepared as previously described [20]. Purification of DC, B cells, natural killer (NK) cells, and CD4^+^ T cells from splenocytes was performed using magnetic beads according to the manufacturer’s instructions (StemSep). Peritoneal exudate macrophages were obtained three days after intraperitoneal injection of 3% sodium thioglycolate medium (2 mL per mouse, Sigma). Cells were seeded into 24 well plates, and after 3 hours, the cells were washed and fresh RPMI medium was added. 5 × 10^5^ splenocytes were seeded in 0.75 ml of heat-inactivated RPMI media, activated with CD3 (2.0 μg/mL) and CD28 (0.5 μg/mL) (CD3/CD28) in the presence or absence of CpG ODN 1668 (1 μg/mL) (CPG) for 72 hours and the IL-10 or IL-30 levels in the supernatant were measured via IL27p28 or IL-10 ELISA) (R&D Systems and eBioscience, respectively). When appropriate, splenocytes were treated with anti-CD40 (10 μg/mL), LPS (1 μg/mL on day 0 and 2), Poly I:C (50 μg/mL), lipoteichoic acid (LTA, 5 μg/mL), rIL12 (50 ng/mL), IFNγ (50 ng/mL), IL-10 (20 ng/mL). Splenocytes depleted of various cell subsets were seeded as mentioned above. For the coincubation assay of CD4 T cells, B cells, DC, whole T cells (CD3^+^), NK cells, and macrophages, 4 × 10^5^ cells of each type were seeded in 500 μL of heat-inactivated RPMI for 72 hours.

### Mice

CD40^-/-^, CD154^-/-^, NF-κB1^-/-^, and IL-10^-/-^ mice were obtained from Jackson Laboratories. C57Bl/6, nude, and SCID mice where purchased from Harlan Laboratories. All experiments were performed using 6–10 week old mice. All the procedures on mice were approved by the IACUC Committee at MD Anderson Cancer Center.

### Flow cytometry analysis

Splenocytes were treated as indicated in the figure, and 72 hours post-incubation, IL-10 expression was analyzed via flow cytometry. Briefly, 4 hours prior to staining, cells were incubated with Brefeldin A. Afterwards, cells were washed in PBS, and stained for CD4 (1:100) or F4/80 (1:100 for 30′ 4°C. After incubation, the cells were permeabilized for 20′ at 4°C, and blocked for FcRs via incubation with anti-CD16 (5 μg/mL) for 20′ at 4°C. After blocking, cells were stained with anti-pERK (1:200), anti- pSTAT3 (1:500), anti-p-p65 (1:1000), or anti-IL-10-Pe antibody for 30′ at 4°C. At last, the cells were washed and analyzed by flow cytometry using Attune (Invitrogen).

### Statistical analysis

Unpaired student’s T test was used to determine significance among groups.

## Abbreviations

Treg: Regulatory T cells; DC: Dendritic cells; IL-10: Interleukin-10; APCs: Antigen presenting cells; ODN: Thiol-modified CpG oligodeoxynucleotide; LTA: Lipoteichoic acid; MFI: Median flouresence intensity.

## Competing interests

The authors declare that they have no competing interests.

## Authors’ contributions

DD performed experiments, designed the experiments, analyzed the results, and wrote the manuscript. SL contributed to the design of the experiments and discussion of the results, and helped in the revision of the manuscript. All authors read and fully approved the final version of the manuscript.

## Supplementary Material

Additional file 1: Figure S1 The combination treatment does not affect the splenocytes proliferation when compared to either treatment alone. Splenocytes treated with CD3/CD28, CpG, or CD3/CD28/CpG for 72 hours and the number of viable cells were measured via trypan blue exclusion. Click here for file

Additional file 2: Figure S2 The combination treatment CD3/CD28/CpG results in a reduction of Treg cells in vitro. Splenocytes treated with CD3/CD28, CpG, or CD3/CD28/CpG for 72 hours and cells were stained for CD4 and FOXP3. Click here for file

Additional file 3: Figure S3 CD3/CD28 and CD3/CD28/CpG induce ERK activation in CD4^+^ T cells. Splenocytes treated with CD3/CD28, CpG, or CD3/CD28/CpG for 10, 30 minutes, or 1 hour. Cells were stained for CD4 and pERK, and the median fluorescence intensity of activated pERK was measured in the gated CD4 positive cells. Click here for file

Additional file 4: Figure S4 IL10 or IL30 have no effect on CD40/CD154 pathway. Splenocytes treated with CD3/CD28/CpG in the presence or absence of recombinant IL10 or IL30. Cells were stained for CD40 and F4/80 or CD154 and CD4. The median fluorescence intensity of CD154 or CD40 was measured in CD4 or F4/80 gated cells, respectively. Click here for file
