# Supplementary figures and images for: The cell-to-cell coordination between activated T cells and CpG-stimulated macrophages synergistically induce elevated levels of IL-10 via NF-κB1, STAT3, and CD40/CD154
Source: Cell Commun Signal. 2013 Dec 13;11:95. doi: 10.1186/1478-811X-11-95 (PMC3880105; doi:10.1186/1478-811X-11-95)

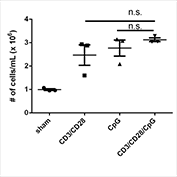

Supplement: Additional file 1: Figure S1 — The combination treatment does not affect the splenocytes proliferation when compared to either treatment alone. Splenocytes treated with CD3/CD28, CpG, or CD3/CD28/CpG for 72 hours and the number of viable cells were measured via trypan blue exclusion. [file 1478-811X-11-95-S1.tiff]
